# Slow Phospholipid Exchange between a Detergent-Solubilized Membrane Protein and Lipid-Detergent Mixed Micelles: Brominated Phospholipids as Tools to Follow Its Kinetics

**DOI:** 10.1371/journal.pone.0170481

**Published:** 2017-01-24

**Authors:** Cédric Montigny, Thibaud Dieudonné, Stéphane Orlowski, José Luis Vázquez-Ibar, Carole Gauron, Dominique Georgin, Sten Lund, Marc le Maire, Jesper V. Møller, Philippe Champeil, Guillaume Lenoir

**Affiliations:** 1 Institute for Integrative Biology of the Cell (I2BC), CEA, CNRS, Université Paris-Sud, Université Paris-Saclay, Gif-sur-Yvette, France; 2 CEA, iBiTec-S, Service de Chimie Bioorganique et de Marquage, Gif-sur-Yvette, France; 3 Medical Research Laboratory, Department of Endocrinology and Internal Medicine, Aarhus University Hospital, Aarhus, Denmark; 4 Centre for Membrane Pumps in Cells and Disease—PUMPKIN, Danish National Research Foundation, Aarhus University, Aarhus, Denmark; Department of Biomedicine, Aarhus University, Aarhus, Denmark; University of Cambridge, UNITED KINGDOM

## Abstract

Membrane proteins are largely dependent for their function on the phospholipids present in their immediate environment, and when they are solubilized by detergent for further study, residual phospholipids are critical, too. Here, brominated phosphatidylcholine, a phospholipid which behaves as an unsaturated phosphatidylcholine, was used to reveal the kinetics of phospholipid exchange or transfer from detergent mixed micelles to the environment of a detergent-solubilized membrane protein, the paradigmatic P-type ATPase SERCA1a, in which Trp residues can experience fluorescence quenching by bromine atoms present on phospholipid alkyl chains in their immediate environment. Using dodecylmaltoside as the detergent, exchange of (brominated) phospholipid was found to be much slower than exchange of detergent under the same conditions, and also much slower than membrane solubilization, the latter being evidenced by light scattering changes. The kinetics of this exchange was strongly dependent on temperature. It was also dependent on the total concentration of the mixed micelles, revealing the major role for such exchange of the collision of detergent micelles with the detergent-solubilized protein. Back-transfer of the brominated phospholipid from the solubilized protein to the detergent micelle was much faster if lipid-free DDM micelles instead of mixed micelles were added for triggering dissociation of brominated phosphatidylcholine from the solubilized protein, or in the additional presence of C_12_E_8_ detergent during exchange, also emphasizing the role of the chemical nature of the micelle/protein interface. This protocol using brominated lipids appears to be valuable for revealing the possibly slow kinetics of phospholipid transfer to or from detergent-solubilized membrane proteins. Independently, continuous recording of the activity of the protein can also be used in some cases to correlate changes in activity with the exchange of a specific phospholipid, as shown here by using the Drs2p/Cdc50p complex, a lipid flippase with specific binding sites for lipids.

## Introduction

It is well recognized that neighbouring lipids are critical for the function of membrane proteins (*e*.*g*. transport or signal transduction), and phospholipids may even be physiological ligands for some of them [1–6]. Detailed study of a membrane protein often requires its solubilization by detergent; it is therefore important to reliably control the lipids remaining in, or introduced into, the detergent- and lipid-containing ‘belt’ which constitutes the immediate environment of the solubilized protein. When trying to delipidate or relipidate such a protein, transfer of lipids between the detergent-solubilized membrane protein and added pure or mixed detergent micelles is often considered as a fairly slow process, probably dependent on the detergent used [7,8]. Yet, to our knowledge, there are only very few reports explicitly measuring the kinetics of this transfer. The possibly slow kinetics of such transfer, especially for experiments performed at low temperature, has indeed been mentioned in early papers devoted to reconstitution procedures, or, more recently, in relation with protein activation by various lipids [9–11], but despite the fact that the kinetics of this exchange has to be taken into account for the design of relevant subsequent experiments, it has more often been alluded to than described and studied in detail.

We here describe a simple method to reveal the kinetics of this exchange, using brominated phosphatidylcholine (BrPC). The membrane protein used was SERCA1a, a P-type calcium-dependent ATPase responsible for Ca^2+^ transport in the sarcoplasmic reticulum lumen and which is the main protein component of sarcoplasmic reticulum membranes. In SERCA1a, twelve tryptophan residues are localized in the transmembrane domain, and their intrinsic fluorescence is sensitive to the presence of bromine atoms on lipid aliphatic chains in their immediate environment [12,13]. The corresponding brominated lipids are considered to mimic unsaturated lipids, as suggested by the facts that liposomes made out of brominated PC behave essentially as liposomes formed with ‘normal’ unsaturated lipid as regards their fluidity properties, and that SERCA1a reconstituted in such brominated PC has an ATPase activity similar to the one in DOPC or egg PC [12]. We here show that exchange of BrPC between dodecylmaltoside (DDM)-solubilized SERCA1a and mixed DDM/egg PC micelles is fairly slow. In particular, it is slower than exchange of the detergent itself, as tested with brominated DDM (which has also been described as a fair analog of DDM [14]), and slower than the membrane solubilization process. We point out the strong dependence of the transfer kinetics on various experimental conditions. Finally, we illustrate the consequences of phospholipid transfer on the activity of two membrane proteins, the above-mentioned Ca^2+^-ATPase SERCA1a and the Drs2p/Cdc50p lipid flippase complex.

## Materials and Methods

### Materials

Brominated phosphatidylcholine (BrPC, 1,2-di-(9,10-dibromo)stearoyl-sn-glycero-3-phosphocholine, #850366) and egg phosphatidylcholine (#840051) were from Avanti Polar Lipids, DDM (n-dodecyl-β-D-maltoside) and C_12_E_8_ (octaethylene glycol monododecyl ether) were from Anatrace, and other chemicals were of standard grade. SERCA1a-containing sarcoplasmic reticulum (SR) membranes (with lipid and protein contents of about 0.5 g lipid per g protein [15]) were prepared from rabbit skeletal muscle as described [16]. Streptavidin-purified yeast Drs2p/Cdc50p complex was also prepared as described [17]. Brominated DDM (BrDDM, 5,6-dibromo-dodecyl-β-D-maltoside) was synthesized as described [18]; its critical micelle concentration (cmc), measured according to the methyl orange method [19], was found only 20% higher than that for DDM (see similar result with 7–8 BrDDM in [14]), and BrDDM affected SERCA1a ATPase activity in a manner very similar to DDM (S1 Fig). Two different buffers were used, either buffer A (100 mM KCl, 1 mM MgCl_2_, 50 mM Tes-Tris at pH 7.5 and 0.1 mM CaCl_2_), or buffer B (100 mM KCl, 5 mM MgCl_2_, 50 mM Mops-Tris at pH 7 and contaminating Ca^2+^ from demineralized water, a few micromolar).

### Tryptophan fluorescence

**Fig 1:**
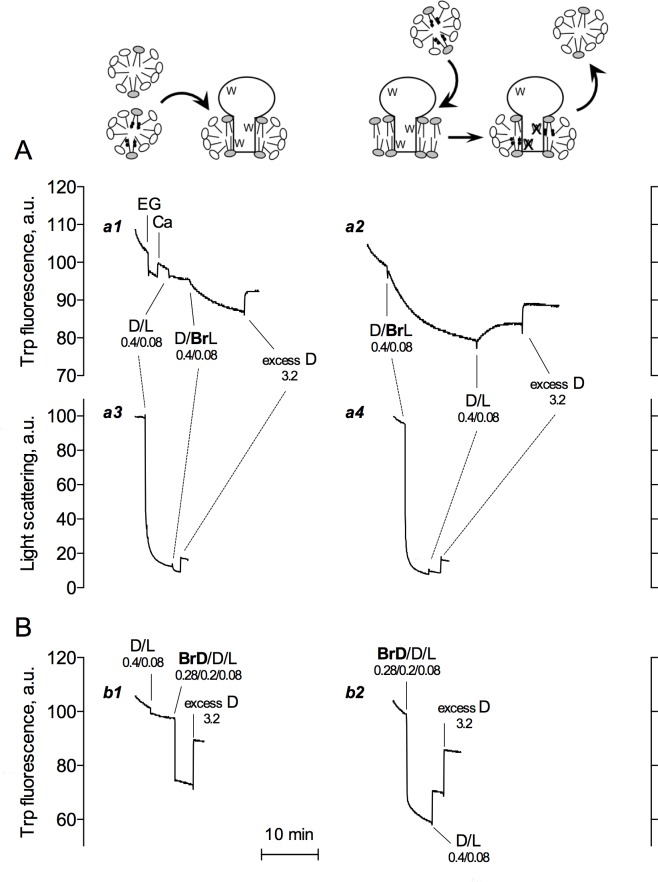
Kinetics of brominated PC exchange between mixed DDM/lipid micelles and SERCA1a solubilized at the same detergent/lipid ratio. (A) Trace *a1*, to 2 mL of SR vesicles suspended at 0.04 mg protein/mL (together with 0.02 mg endogenous lipid/mL) in buffer A at 20°C, 0.6 mM EGTA (EG) followed by 0.6 mM CaCl_2_ (Ca) were first added as controls, to illustrate the classical Ca^2+^-dependent changes in SERCA1a intrinsic fluorescence as well as the usual down-drift in fluorescence intensity accompanying such measurements (see Materials and Methods). SERCA1a was then solubilized by adding mixed DDM/egg PC micelles (D/L, containing the unbrominated lipid at a ratio of 5 g detergent/g lipid, i.e. ~7 mol/mol) at final concentrations of 0.4 and 0.08 mg/mL for DDM and egg PC, respectively. Mixed micelles with brominated lipids (D/BrL) were then added and this was followed by addition of excess DDM (to reach a final total DDM concentration of 4 mg/mL). For Trace *a2*, additions were performed first with brominated (D/BrL) and then with unbrominated (D/L) lipid. Traces *a3* and *a4* correspond to experiments similar to those in *a1* and *a2*, respectively, but now measuring light scattering at 290 nm. (B) In Traces *b1* and *b2*, unbrominated lipid was used but DDM was replaced by 5,6-brominated DDM (BrD) at the same molar concentration. Traces here have not been corrected for dilution effects. This dilution effect only becomes somewhat significant when adding excess DDM at 3.2 mg/mL (32 μL of a 200 mg/mL stock solution resulting in a 1.6% dilution). Numbers indicate the concentrations of detergent and lipid added at each step to the cuvette, in mg/mL. The cartoon on top depicts the principle of the experiment. Lipids are represented with a grey headgroup, detergent molecules are represented with a white headgroup and bromine ions are displayed as black dots. Each trace corresponds to one experiment representative of three independent experiments.

Fluorescence measurements (with excitation and emission wavelengths of 290 and 330 nm) and light scattering measurements (at 290 nm) were performed on a Fluorolog spectrofluorometer (Horiba). In the fluorometer stirred cuvette, sarcoplasmic reticulum vesicles were suspended at 40 μg protein/mL (together with ~20 μg endogenous lipid/mL) in 2 mL buffer A at 20°C or in 2 mL buffer B at various temperatures. As preliminary controls, we first monitored the classical fluorescence changes (by a few % of the initial fluorescence, see e.g. [20]) observable upon initial addition to these membranes of a calcium chelator (0.6 mM EGTA) and upon subsequent addition of an equivalent amount of calcium (0.6 mM Ca^2+^), as well as the usual photobleaching of tryptophan residues (together with the accompanying slow vesicle adsorption onto the cuvette’s walls). Small volumes of detergent or lipid together with detergent were then added from various stock solutions of detergent micelles or mixed micelles. For instance, for Fig 1, these stock solutions (in water) were DDM/egg PC (D/L), 50 mg/mL and 10 mg/mL, respectively (or, in one case, 50 mg/mL and 20 mg/mL, respectively); DDM/BrPC (D/BrL), 50 mg/mL and 10 mg/mL, respectively; DDM alone (D), 50 mg/mL or 200 mg/mL depending on experiments; brominated DDM alone (BrD), 70 mg/mL (corresponding to about the same molar concentration, ~100 mM, as in 50 mg/ml of the unbrominated DDM); a DDM/lipid stock solution at 50 mg/mL and 20 mg/mL, respectively, was also prepared. In the traces shown, the small intensity changes due to mere dilution (by 2% at most) of the protein sample upon the various additions have not been corrected for.

### ATPase activity

The ATPase activity of SERCA1a was assayed using a coupled-enzyme assay [21], using buffer A supplemented with 5 mM MgATP and 0.05 mM EGTA (hence, because of the 0.1 mM CaCl_2_, 50 μM excess Ca^2+^) as well as 50 μg/mL pyruvate kinase, 100 μg/mL lactate dehydrogenase, 1 mM phosphoenolpyruvate and about 0.3 mM NADH, which allows continuous monitoring of the ATPase activity of the sample by following the concomitant drop in NADH absorption in a diode array spectrophotometer (HP-Agilent 8453). A cut-off filter (MTO J310A) was used to minimize photobleaching of NADH. For measurement of the ATPase activity of the Drs2p/Cdc50p flippase, the purified yeast Drs2p/Cdc50p complex at a concentration of 0.12 mg protein/mL) [17], had first been treated with 4.5 μg/mL chymotrypsin for 60 minutes at 20°C in a medium consisting of buffer B supplemented with 20% glycerol, 1 mg/mL DDM, 0.05 mg/mL POPS and 0.025 mg/mL PI4P (Azouaoui et al, manuscript in preparation). In the spectrophotometer cuvette, it was then diluted 180-fold into buffer B at 30°C supplemented with 20% glycerol, 0.8 mg/mL DDM and 0.06 mg/mL POPS, either in the additional presence of 0.025 mg/mL PI4P from the start or in its absence, and also containing 1 mM MgATP as well as the above-listed components of the coupled-enzyme assay. PI4P and egg PC were added to the spectrophotometer cuvette after more than half an hour of continuous recording in the absence of PI4P of the slowly declining sample activity.

## Results

### Lipid-exchange between DDM-solubilized SERCA1a and mixed micelles is slow

The experiments illustrated in Fig 1 were designed to reveal the kinetics of phosphatidylcholine (PC) exchange between mixed DDM/lipid micelles and the lipid/detergent belt around DDM-solubilized SERCA1a, making use of the ability of bromine-containing phospholipids to quench the tryptophan fluorescence of the protein when they are close enough.

For control purposes, the initial portion of Trace *a1* in Fig 1 shows the Ca^2+^-dependent fluorescence changes experienced by SERCA1a when Ca^2+^ is chelated by the Ca^2+^ chelator EGTA or subsequently added back (about 5% changes), as well as the classical down-drift in fluorescence intensity accompanying such measurements. This drift is partly due to Trp photobleaching, and partly to slow protein adsorption onto the cuvette’s walls. Trace *a1* also shows what happens when mixed detergent/lipid micelles (D/L) containing unbrominated lipids, here egg PC (at a ratio of 5 g detergent/g lipid), are added to SERCA1a-containing membranes. Trp fluorescence first drops, only by a few %, and subsequently slowly rises back slowly, suggesting slow kinetics for reaching equilibrium of this protein/detergent/lipid system. These changes have already been observed previously but not studied in detail [13], because of their small amplitude (which makes them sensitive to aggregates or bubbles passing in the beam-illuminated region of the cuvette) and slow kinetics (which make them not always easily distinguishable from photobleaching). At this step, concentrations of DDM and egg PC were 0.4 mg/mL and 0.08 mg/mL, respectively, and the SERCA1a-containing membranes were indeed solubilized, as confirmed by light scattering measurements (Fig 1A, Trace *a3*). Assuming that the concentration of monomeric DDM is ~0.08 mg/mL (the cmc of DDM in the presence of lipid [13]), and considering the only modest amount of DDM required for solubilizing the SR membranes at 0.04 mg/mL of protein and ~0.02 mg/mL of endogenous lipid (less than 1 g detergent/g lipid), this leaves most of the detergent and egg PC in the fluorometer cuvette as mixed micelles. When a second addition of mixed micelles was performed, now with BrPC-containing mixed micelles (D/BrL), the fluorescence signal dropped markedly (by about 10%) and remarkably slowly, over a few minutes. This drop was rapidly reversed when lipid-free excess detergent was added (Fig 1A, Trace *a1*), resulting in a final total concentration of DDM of 4 mg/mL and a detergent/lipid w/w ratio 5-fold higher than the initial one. This final addition of a large amount of micelles was accompanied by a small increase in light scattering, due to scattering by the micelles themselves (Fig 1A, Trace *a3*). Judging from previous work, the BrPC-induced fluorescence drop must have been due to quenching of Trp residues in the SERCA1a transmembrane region by brominated lipids coming in their close vicinity, while its reversal upon addition of excess DDM must reflect the strong, although possibly not complete, DDM-induced delipidation resulting from a high detergent/lipid ratio [12,13].

Trace *a2* in Fig 1A illustrates the inverse experiment where the first addition was of DDM/BrPC micelles (D/BrL), and the second with DDM/egg PC micelles (D/L). The first addition (D/BrL) still triggered rapid solubilization (see Trace *a4*), but Trp fluorescence quenching by the added BrPC remained fairly slow (half-time, ca 3 min), with a total amplitude larger (about 20%) than in Trace *a1*, in line with the larger BrPC to total lipid and detergent ratio. When a second addition, now with unbrominated egg PC, was performed (D/L), partial back exchange of BrPC for egg PC resulted in the expected partial recovery of fluorescence, up to a level approximatively similar to what was seen in Trace *a1* under the same final conditions. This recovery again took place fairly slowly, at about the same rate as when BrPC was exchanged in the other direction in Trace *a1*. At the end, addition of excess DDM micelles triggered more complete fluorescence recovery, up to about the same level as in Trace *a1*, as expected.

In the last two traces of Fig 1, we verified that detergent exchange was fast under all conditions, as generally assumed for DDM in the case of interaction with membrane proteins (e.g. Fig 7 in [13]). For this purpose, mixed micelles containing brominated detergent together with unbrominated egg PC were used (BrD/L). Traces *b1* and *b2* in Fig 1B show that a rapid fluorescence drop was indeed observed upon addition of BrD/L to either already solubilized SERCA1a or intact membranes, even faster than the kinetics of solubilization followed by light scattering in Traces *a3* and *a4*. Similar results were obtained using two other previously described brominated alkylmaltoside detergents, 7,8-BrDDM [14] or 10,11-BrUDM (10,11-dibromo-β-D-undecylmaltoside, [22]), instead of the 5,6-BrDDM species used here (not shown).

### Effect of temperature and micelle concentration on exchange rates

**Fig 2:**
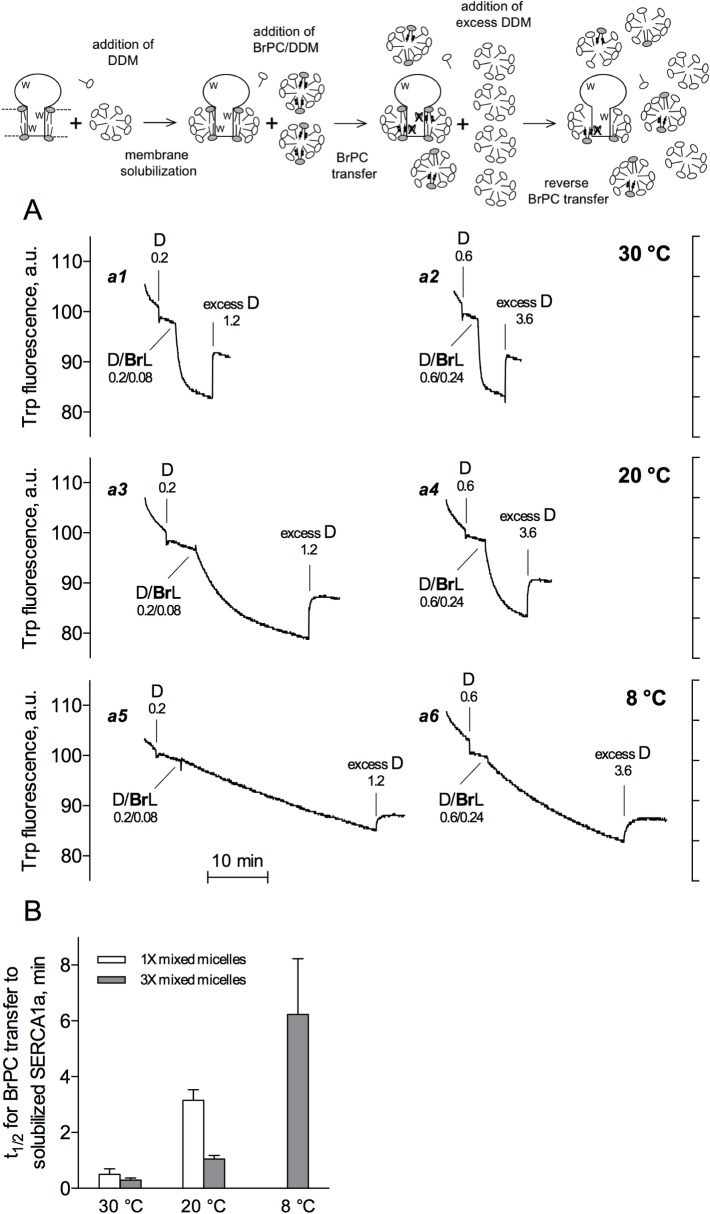
Kinetics of BrPC exchange in the presence of different amounts of the same mixed micelles, and at various temperatures. For these experiments, buffer B was used. (A) For Traces *a1* and *a2*, the temperature was 30°C. To SR vesicles at 0.04 mg protein/mL and 0.02 mg endogenous lipid/mL, DDM was initially added at a concentration of either 0.2 mg/mL (Trace *a1*) or 0.6 mg/mL (Trace *a2*), resulting in membrane solubilization in both cases. Then BrPC in DDM was added, either 0.08 mg/mL in 0.2 mg/mL (Trace *a1*) or 0.24 mg/mL in 0.6 mg/mL (Trace *a2*). The total DDM concentrations therefore were 0.4 or 1.2 mg/mL, but the BrPC/DDM ratio remained the same. At the end, ‘excess’ DDM was added, at 1.2 or 3.6 mg/ml. The final total DDM concentrations therefore were 1.6 or 4.8 mg/mL. Traces *a3* and *a4*, same as *a1* and *a2* but the temperature was 20°C. Traces *a5* and *a6*, same as *a1* and *a2* but the temperature was 8°C. Traces have not been corrected for dilution effects. In the case of Traces *a2*, *a4*, and *a6*, this dilution effect only becomes somewhat significant when adding excess DDM at 3.6 mg/mL (36 μL of a 200 mg/mL stock solution resulting in a 1.8% dilution). The cartoon on top depicts the principle of the experiment. Each trace corresponds to one experiment representative of three to four independent experiments. Numbers in panel A indicate the concentrations of detergent and lipid added to the cuvette at each step, in mg/mL. (B) Half-times for BrPC exchange calculated from traces displayed in (A). Note that when experiments were performed at 8°C and in the presence of mixed D/BrL micelles at only a 1X concentration (Trace *a5*), because of the much slower rate of phospholipid exchange at this temperature, we could not reliably extract rate constants for the exchange process. Data are presented as the mean ± S.D. (error bars) of three to four independent experiments.

In Fig 2 experiments, we focused on the transfer of BrPC from mixed micelles to SERCA1a previously solubilized with micelles of DDM only. In these experiments, BrPC transfer was found to be highly temperature-dependent, with half-times ranging from less than 1 minute at 30°C (Traces *a1* and *a2*, and Fig 2B) to more than 6 minutes at 8°C (Trace *a5* and *a6*, and Fig 2B). At low temperature, the very slow quenching of Trp fluorescence by BrPC was in fact not so easily distinguished from mere spontaneous photobleaching, and we therefore could not reliably extract rate constants for the exchange process at 8°C. But this exchange did occur, as testified by its reversal upon addition of excess DDM.

Remarkably, transfer of BrPC was significantly faster when 3-fold higher total concentrations of the same micelles and mixed micelles were used, keeping the same lipid to detergent ratio to deliver BrPC (compare Traces *a2* with *a1*, *a4* with *a3*, and *a6* with *a5* in Fig 2A). This strongly suggests that phospholipid exchange mainly occurs thanks to collisions of the mixed micelles with the detergent-solubilized protein, and not merely thanks to the exchange of lipid monomers through the water phase, because the small concentration of these lipid monomers in the water phase should remain the same in the presence of different concentrations of mixed micelles, just like the concentration of monomeric detergent in the presence of different concentrations of total detergent above the cmc.

**Fig 3:**
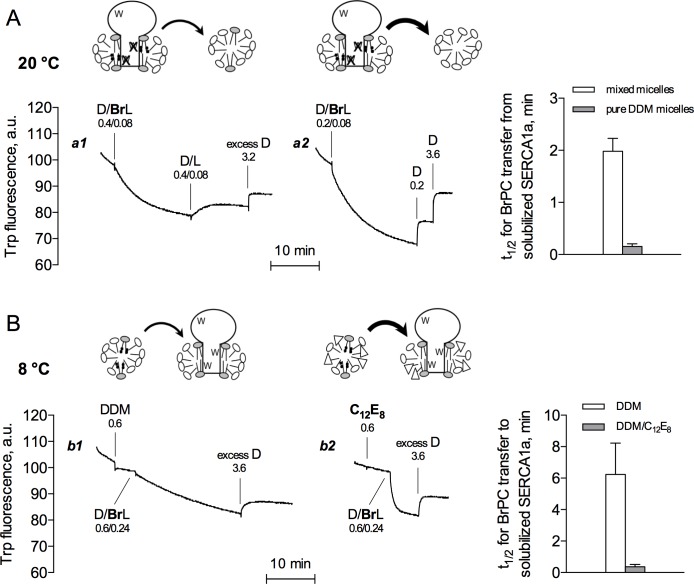
BrPC transfer is greatly accelerated during asymmetrical transfer, as well as in the additional presence of C_12_E_8_. (A) Temperature was 20°C and buffer A was used, as for Fig 1. Each trace corresponds to one experiment representative of three independent experiments. Trace *a1* illustrates an experiment similar to that previously shown as Trace *a2* of Fig 1 (identical assay conditions but independent experiments). Trace *a2* shows a related experiment, where brominated lipid was first added at a higher bromolipid to detergent ratio, as indicated, while subsequently only DDM was added, in two steps, to monitor the kinetics of fluorescence recovery under ‘asymmetrical’ conditions. The histograms on the right represent half-times for BrPC transfer from solubilized SERCA1a to either mixed egg PC/DDM micelles (open bars, as in Trace *a1*) or pure DDM micelles (grey bars, as in Trace *a2*). Data are presented as the mean ± S.D. (error bars) of three independent experiments. (B) Temperature was here 8°C and buffer B was used. Each trace corresponds to one experiment representative of three independent experiments. Trace *b1* illustrates an experiment similar to that previously shown as Trace *a6* of Fig 2 (identical assay conditions but independent experiment). Trace *b2* shows a related experiment, where membranes were initially solubilized with C_12_E_8_ at 0.6 mg/mL instead of DDM. Then BrPC in DDM was added (at 0.24 mg/mL and 0.6 mg/mL, respectively), as for the left trace. Phospholipid exchange therefore now took place in the presence of 0.24 mg/mL BrPC, 0.6 mg/mL DDM and 0.6 mg/mL C_12_E_8_ instead of 0.24 mg/mL BrPC and 1.2 mg/mL DDM. Since detergent itself is thought to exchange rapidly, DDM and C_12_E_8_ presumably soon exchanged with each other, hence the cartoon on the right representing a mixed ternary micelle and a protein belt with both detergents. In this cartoon, the polar headgroup of C_12_E_8_ is represented as triangles. At the end, excess DDM was added at 3.6 mg/ml, so that total DDM and C_12_E_8_ concentrations were 4.2 and 0.6 mg/mL, respectively. Numbers indicate the concentrations of detergent and lipid added to the cuvette at each step, in mg/mL. The histograms on the right represent half-times for BrPC transfer to solubilized SERCA1a calculated from traces of the same panel. Data are presented as the mean ± S.D. (error bars) of three independent experiments.

In both Figs 1 and 2, recovery of Trp fluorescence upon final addition of a large excess of DDM was fairly fast. One conceivable reason for this is that DDM micelles were added in fairly large amounts at this step. But this was also the case after adding DDM at a lower concentration, as shown in Trace *a2* of Fig 3A. In this experiment, to reverse the BrPC-induced drop in fluorescence intensity, pure DDM micelles were added at rather low concentrations (Trace *a2* in Fig 3A), instead of adding mixed micelles with unbrominated lipids (Trace *a1* in Fig 3A). The kinetics of recovery was about 5-fold faster (see histograms in Fig 3A), suggesting that transfer of BrPC from solubilized SERCA1a surrounded by a significant amount of lipid to lipid-free DDM micelles (as in Trace *a2*) is significantly faster than exchange with lipids on both sides (as in Trace *a1*). Kinetics of phospholipid transfer under different ‘asymmetrical’ conditions have already been studied and found different from kinetics under ‘symmetrical’ conditions; in that case, transfer of phosphatidylcholine between sonicated vesicles made of lipids with either identical or different chain lengths had been studied [23]. The experiment illustrated in Trace *a2* of Fig 3A is another example of such transfer in an asymmetrical context.

Along a related line, Panel B in Fig 3 shows that BrPC transfer from BrPC-containing mixed micelles to the immediate environment of solubilized SERCA1a was greatly accelerated if the SERCA1a-containing membranes had been initially solubilized by C_12_E_8_, a detergent which has the same hydrophobic tail as DDM but a different polar headgroup (Trace *b2* versus Trace *b1* and histograms in Fig 3B). DDM and C_12_E_8_ both have low cmc values in the absence of lipids, 0.1 mg/mL and 0.05 mg/mL, respectively, and aggregation numbers which are not very different, 110–140 and 90–120 monomers per micelle, respectively [24]. Note that the cartoon on top of Trace *b2* in Fig 3B assumes that exchange of C_12_E_8_ and DDM between the SERCA1a detergent belt and the added mixed micelles has already occurred rapidly, and resulted in mixing of the two detergents, as suggested by the experiments with BrDDM (Fig 1) and by the fact that C_12_E_8_ is a detergent interacting with lipids even faster than BrDDM [13]. When excess DDM was added, fluorescence recovery, and hence BrPC transfer, was also faster in the presence of C_12_E_8_. At variance, we found that initial membrane solubilization by Lauryl Maltose Neopentyl Glycol (LMNG), a kind of DDM dimer [25], slowed down subsequent BrPC transfer (not shown). These results illustrated in Fig 3 reveal that the kinetics of phospholipid transfer does not only depend on the frequency of the collisions between the protein and the detergent micelles around, but that the chemical nature of the micellar interface also matters.

### Phospholipid dissociation as deduced from functional assays

**Fig 4:**
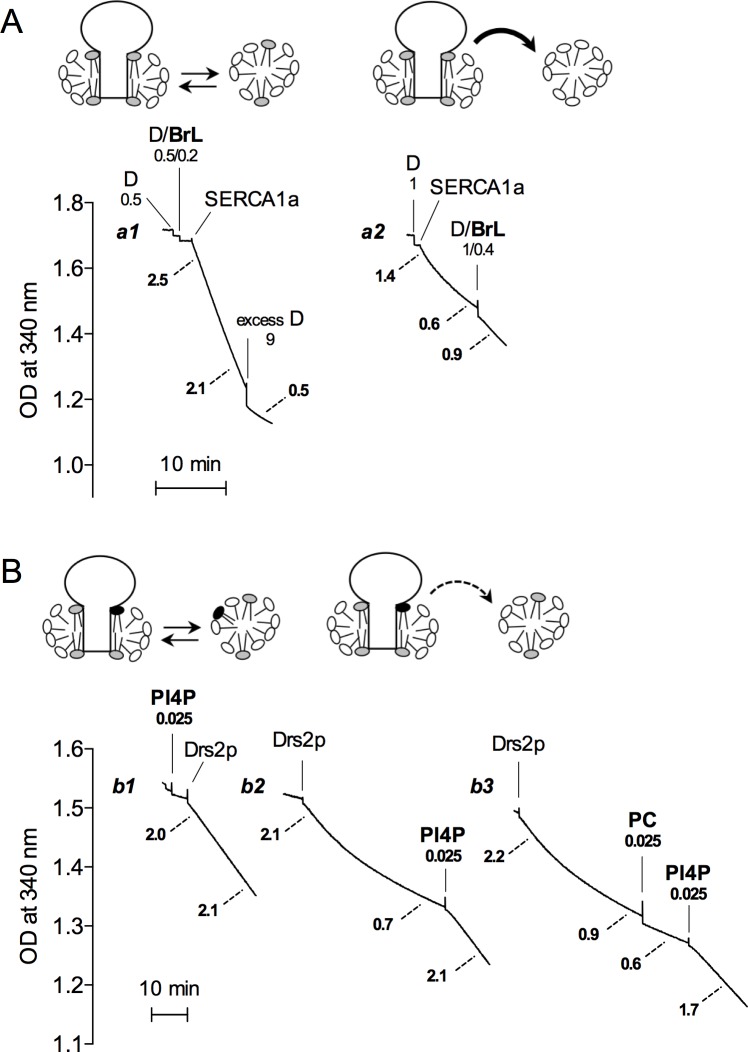
Slow phospholipid transfer can also be detected by functional measurements. (A) ATPase activity measurements with SERCA1a. SERCA1a at 2 mg protein/mL was solubilized with mixed micelles at 10 mg/mL DDM and 2 mg/mL BrPC. This solubilized enzyme was diluted 500-fold into either (Trace *a1*) buffer A to which a total of 1 mg/mL DDM and 0.2 mg/mL BrPC had been added (and excess DDM at 9 mg/mL was added at the end), or (Trace *a2*) buffer A to which 1 mg/mL DDM only had been added. In the latter case, mixed micelles were added after some time, resulting in final concentrations of detergent and lipid twice higher than those in Trace *a1* (2 mg/mL DDM and 0.4 mg/mL BrPC), but at the same detergent/lipid ratio. ATPase activity was measured at 20°C. The cartoon on top depicts the principle of the experiment. Lipids and detergent molecules are represented with grey and white headgroups, respectively. (B) ATPase activity measurements with purified and chymotrypsin-treated Drs2p/Cdc50p complex. To be able to reveal the ATPase activity of the complex using the enzyme-coupled assay, we first subjected the purified Drs2p/Cdc50p to limited proteolysis with chymotrypsin in the presence of DDM, PS and PI4P (see Materials and Methods). This treatment relieves auto-inhibition of Drs2p/Cdc50p, increasing significantly its ATP hydrolysis rate (manuscript in preparation). The protein concentration of the purified and proteolyzed sample was 0.12 mg/mL. After dilution, its ATPase activity was assayed at 30°C in buffer B, supplemented with 20% glycerol as well as with 0.8 mg/mL DDM and 0.06 mg/mL POPS. For Trace *b1*, this medium was also supplemented with 0.025 mg/mL PI4P and with 0.25 mg/mL DDM, and the chymotrypsin-treated Drs2p/Cdc50p sample was then added, with 180-fold dilution. For Trace *b2*, the Drs2p/Cdc50p sample was directly added, and 0.025 mg/mL PI4P together with 0.25 mg/mL DDM was only added after a few tens of minutes. For Trace *b3*, after adding the Drs2p/Cdc50p sample, 0.025 mg/mL egg PC (PC) was first added together with 0.25 mg/mL DDM, and 0.025 mg/mL PI4P together with 0.25 mg/mL DDM was subsequently added. The cartoon on the left depicts the principle of the experiment illustrated by Trace *b1* while the cartoon on the right depicts the principle of the experiments illustrated by Traces *b2* and *b3*. PI4P is represented with a black headgroup while POPS is represented with a grey headgroup. Numbers at the edge of dotted lines represent the specific activity (in μmol.min^-1^.mg^-1^) calculated from the slopes of the corresponding traces. Each trace corresponds to one experiment representative of two to three independent experiments.

We finally took benefit of the previous observation that delipidation of SERCA1a by excess DDM leads to a distinct reduction of its ATPase activity (e.g. Fig 8B in [13]) to investigate the possibly slow kinetics of phospholipid exchange while continuously monitoring the activity of detergent-solubilized SERCA1a. In Fig 4A, SERCA1a-containing membranes were first solubilized with DDM/BrPC mixed micelles under conditions (2 mg/mL protein, 10 mg/mL DDM, 2 mg/mL BrPC) presumably resulting in only modest delipidation. When this enzyme was diluted into an assay medium containing both DDM and BrPC (at 1 mg/mL and 0.2 mg/mL, respectively), there was virtually no change in its activity over 10 minutes, while subsequent addition of excess DDM (total DDM concentration was 10 mg/mL) and the expected concomitant delipidation did slow down this activity (Trace *a1* in Fig 4A). In contrast, when solubilized SERCA1a was diluted into an assay medium containing DDM alone at the same total concentration (Trace *a2* in Fig 4A), its activity significantly diminished over time during the first few minutes (down to 0.6 μmol.min^-1^.mg^-1^: the corresponding region of Trace *a2* is no longer a straight line). However, the observed reduction in activity was not fully reversed upon addition of lipids up to the same detergent/BrPC ratio (D/BrL of 2/0.4 or 1/0.2 in Traces *a2* and *a1* of Fig 4A, respectively), implying that part of this reduction in activity was due to slow and irreversible detergent-induced inactivation of solubilized SERCA1a during its turnover in the absence of protecting lipids. The existence of such irreversible inactivation has been previously documented in [21] for a number of detergents, including DDM for which the half-time of this inactivation was of the order of 10 min (‘*k*_*TO*_’ = 0.07 min^-1^ in Table I of [21]). Because of this irreversible inactivation process, the reduction in activity in Trace *a2* can therefore not be unambiguously interpreted as resulting from the sole delipidation, and does not *per se* allow to reliably measure the exact kinetics of this delipidation during turn-over.

A more informative result was obtained using a related membrane protein, the Drs2p/Cdc50p lipid flippase complex, the ATPase activity of which is strictly dependent on phosphatidylserine (PS), the lipid transported by the flippase, and is functionally regulated by phosphatidylinositol-4-phosphate (PI4P), which binds to Drs2p with high affinity [17]. For these experiments, this enzyme had been mildly treated with chymotrypsin for enhancing its activity. Indeed, chymotrypsin trims off regions of the Drs2p chain that are predicted to be intrinsically disordered and that act as auto-inhibitory regions (unpublished observations and [26]). When this solubilized truncated enzyme, prepared in the presence of DDM, PS and PI4P, was diluted into an assay medium also containing DDM, PS and PI4P, it retained a high activity (~2 μmol ATP hydrolyzed.min^-1^.mg^-1^) over more than 20 minutes (Fig 4B, Trace *b1*). In contrast, when the enzyme was diluted into an assay medium containing DDM and PS only, its ATPase activity slowly diminished over more than half an hour at 30°C, before reaching about 20% only of its initial value (Fig 4B, Trace *b2*). In this case, remarkably, full recovery of its initial activity could be achieved by subsequent addition of PI4P (Fig 4B, Trace *b2*). In addition, recovery of the initial activity was specific of PI4P, as neither egg PC (Fig 4B, Trace *b3*) nor phosphatidylethanolamine (data not shown) could restore ATPase activity. From these results, we conclude that the slow kinetics of the decrease in activity was fully due to dissociation of the regulatory lipid PI4P, with no concomitant irreversible inactivation. The high-affinity binding of PI4P to Drs2p therefore results in a particularly slow overall dissociation rate for PI4P (with a half-time for PI4P dissociation of about 10–15 minutes), even at 30°C.

## Discussion

Here, using a brominated phospholipid, we describe a convenient and hopefully widely applicable method to reveal the kinetics of lipid exchange to or from the immediate environment of a detergent-solubilized membrane protein (Fig 1). The kinetics of brominated phospholipid exchange proved not only to strongly depend on temperature, but also to depend on the number of mixed micelles added to trigger this exchange (Fig 2). The latter finding suggests a major role for the collisions of the detergent-solubilized protein with the detergent mixed micelles providing brominated lipid, or with the lipid-free micelles added to remove brominated lipid from the vicinity of the protein. It supports the general view that because of their very low solubility in water, lipids can probably only reach the lipid-detergent belt around the protein (or leave it, in reverse experiments) after collision of the detergent-solubilized protein with detergent micelles, which will either deliver these lipids to the surrounding of the protein, or accept them.

Elucidating the detailed scenario of what happens during such collision is nevertheless outside the scope of the present report. Worth noting, however, is the fact that the kinetics of phospholipid transfer appears to be significantly altered under ‘asymmetrical’ conditions, or in the additional presence of other detergents (Fig 3): both facts suggest that the kinetics of phospholipid exchange does not solely depend on the frequency of collisions between the solubilized protein and the micelles around, but that the efficiency of each collision also depends on the chemical nature of the interface. In relation with the particular effect of C_12_E_8_, note that this detergent has already been found to be more efficient than DDM for delipidation of SERCA1a during size-exclusion chromatography [27], while the opposite was true for the human erythrocyte anion-exchanger, indicating that the relative efficiency of a detergent for delipidating a particular protein may also depend on the protein [8].

It would also be of interest to know whether different phospholipids (*e*.*g*. PS, or PE) display different exchange rates, to further document the possible influence of polar headgroups at the interface between two colliding partners on the efficiency of phospholipid exchange during collision. Checking this would only require to prepare the corresponding brominated lipids (starting from unsaturated lipids), something which has already been described for a variety of phospholipid head groups [28,29]. Future work will hopefully achieve this.

Previous studies have already addressed the kinetics of phospholipid exchange between bilayers in the absence or presence of fusogens [23,30], the kinetics of exchange between model lipoproteins [31], the kinetics of exchange of lysophospholipids between detergent micelles and liposomes [32,33], the kinetics of surfactant or polymer exchange between micelles [34,35], the kinetics of phospholipid transfer between certain mixed micelles [36,37], or even the dynamics of membrane protein/amphipol association [38]. Yet, to our knowledge, only one old report [9] has reported the time-dependent consequences of phospholipid transfer between mixed micelles and solubilized proteins, despite the obvious significance of such studies for proper handling of fragile detergent-solubilized membrane proteins. Our work will therefore hopefully contribute to filling this gap by providing a simple method to follow the kinetics of such exchange.

Unexpectedly, the transfer kinetics of the brominated phospholipid into the immediate environment of SERCA1a proved to be fairly similar when starting from intact membranes or when starting from already solubilized material (compare fluorescence drops in Traces *a2* and *a1* of Fig 1, respectively). This is presumably because solubilization, as deduced from light scattering in Trace *a4*, was much faster than lipid exchange and therefore hardly interfered with this subsequent lipid exchange. Solubilization itself was much faster probably because detergent *monomers* play the major role in solubilization [24]. An additional comment about this is that what is usually called ‘solubilization’ (as deduced from centrifugation experiments, or from light scattering measurements, or merely seen by eye as a clarification process) only corresponds to detergent-induced dissociation of the membrane into smaller objects, the turbidity of which is much smaller than that of the original membranes. The rapid kinetics observed in light scattering measurements only reflects these rapid detergent-induced size changes, before slower equilibration of lipids takes place, and the slow subsequent step of delipidation only marginally influences the light scattering properties of these already small ‘solubilized’ objects. The final minor slow phases in the observed light scattering drops illustrated in Traces *a3* and *a4* possibly reflect in part this delayed delipidation.

Finally, it should be mentioned that the above results have been presented as if lipids surrounding solubilized SERCA1a were constituting a homogenous population of annular, ‘non-sticky’ [39] lipids, associated with the protein in a non-specific manner, and therefore free to ‘jump’ towards the micelles colliding with the protein. This is obviously an oversimplification, but in the case of SERCA1a it is probably a reasonable first approximation, which has found recent support in molecular dynamics simulation experiments [40]. In the various SERCA1a crystals analyzed to date, a few PC molecules can be resolved, and yet, sites for them do not seem to have strong specificity as regards their alkyl chains [5]. The situation is somewhat more blurred regarding the effect of lipid headgroups, but although anionic lipids may modulate the activity for SERCA1a [41], such modulation remains moderate and seems not to be due to actual non-annular lipid binding to specific sites [28]. Most lipids around SERCA1a are therefore probably fairly free to exchange. In contrast, if a particular protein presents a specific binding site for a particular lipid, strong (although possibly dynamic) binding of this lipid to the protein will necessarily slow down the overall jump of the lipid from the lipid/detergent belt around the protein into surrounding lipid-free detergent micelles. For the Drs2p/Cdc50p complex, this is most probably the case for the regulatory lipid PI4P, whose dissociation from Drs2p/Cdc50p is remarkably slow, as deduced from ATPase measurements (Fig 4B). In such cases, brominated lipids will probably reveal more complicated exchange patterns, and synthesis of brominated lipids with headgroups different from choline will be even more desirable.

To examine whether brominated phospholipids can indeed be used as tools with a large range of membrane proteins, one has to consider the mechanisms which have been proposed to result in bromine-dependent quenching. Quenching of Trp fluorescence by dibrominated compounds now appears to be much more efficient than what was expected from the close contact-dependent quenching by mono-brominated compounds originally considered (and discussed in [12,14,42–44] for quenching by either brominated or spin-labelled lipids), and authors now state [43,44] that brominated lipids should more appropriately be defined as short-range quenchers, involving some sort of dipole-dipole interaction (with an R_0_ close to 8–10 Å), rather than as strictly contact quenchers, so that Trp residues are likely to be able to report on the presence of bromolipids even if they are not exactly located ‘in front’ of the bromines. However, depending on whether the reporter Trp residue is located close to a specific binding site for a particular lipid, or far from it, it will be more sensitive to occupancy of this site by the bromolipid, or more sensitive to the mere lipid presence in the annulus [45], and the kinetic approach described here will mainly report either on the non-annular lipids, or on the annular ones. In such cases, precisely monitoring the possibly slow exchange of critical lipids might need special introduction by site-directed mutagenesis of a tryptophan residue close to the desired site, as has been previously done to study the static lipid-protein interactions for the mechanosensitive channel MscL [45]. Alternatively, functional measurements, for instance as illustrated in Fig 4B, might be used to reveal exchange of lipids at those specific sites. Combining the two approaches should allow to study a large variety of lipid-sensitive membrane proteins.

## Supporting Information

S1 Fig *5*,*6-*Bromination of the detergent chain does not greatly affect the basic properties of DDM. (A) Detergent cmc (arrows), as deduced from the differential spectrum of methyl orange (40 μM) observed in the presence of increasing concentrations of detergent. (B) Perturbation and solubilization of SR vesicles (4 μg/mL) by detergent, as deduced from 90° light scattering measurements at 290 nm. (C) Alteration by detergent of the ATPase activity of ionophore-treated SR vesicles (4 μg/mL SR, 1 μg/mL calcimycine). 0.1 mM Ca^2+^ and 0.05 mM EGTA were present in buffer A for Panels B and C, together with 5 mM MgATP and a regenerating system for Panel C. Closed symbols correspond to brominated DDM, open symbols correspond to unbrominated DDM. The latter results are similar to those in Fig 8B of [13].
(TIF)
