# Supplementary figures and images for: Slow Phospholipid Exchange between a Detergent-Solubilized Membrane Protein and Lipid-Detergent Mixed Micelles: Brominated Phospholipids as Tools to Follow Its Kinetics
Source: PLoS One. 2017 Jan 24;12(1):e0170481. doi: 10.1371/journal.pone.0170481 (PMC5261732; doi:10.1371/journal.pone.0170481)

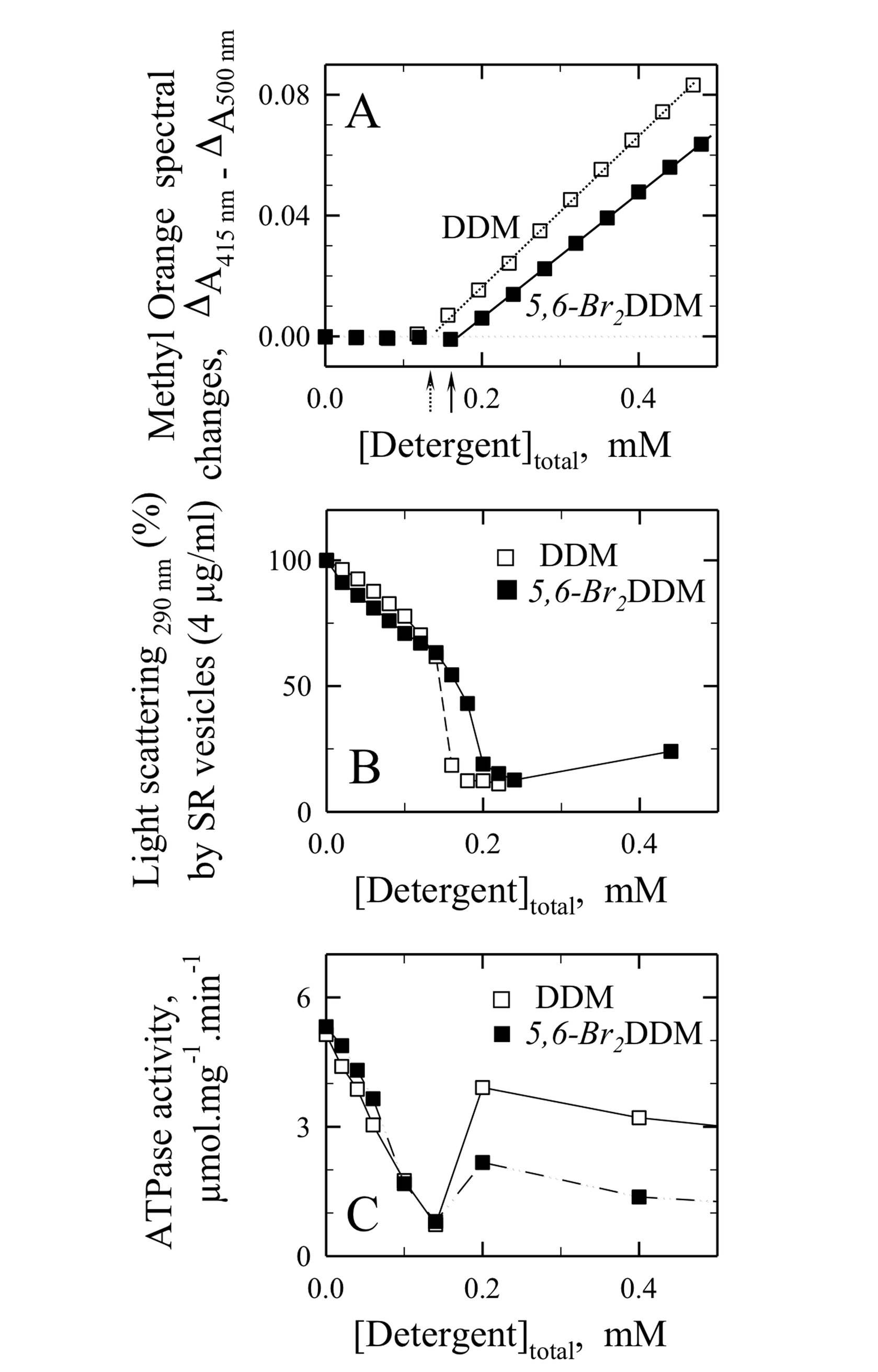

Supplement: S1 Fig — (A) Detergent cmc (arrows), as deduced from the differential spectrum of methyl orange (40 μM) observed in the presence of increasing concentrations of detergent. (B) Perturbation and solubilization of SR vesicles (4 μg/mL) by detergent, as deduced from 90° light scattering measurements at 290 nm. (C) Alteration by detergent of the ATPase activity of ionophore-treated SR vesicles (4 μg/mL SR, 1 μg/mL calcimycine). 0.1 mM Ca2+ and 0.05 mM EGTA were present in buffer A for Panels B and C, together with 5 mM MgATP and a regenerating system for Panel C. Closed symbols correspond to brominated DDM, open symbols correspond to unbrominated DDM. The latter results are similar to those in Fig 8B of [13]. (TIF) [file pone.0170481.s001.tif]
